# P-548. Detection of Recombinant Parechovirus-A5 in Neonatal Sepsis-like Illness and its Community Circulation Confirmed by Wastewater Surveillance

**DOI:** 10.1093/ofid/ofaf695.763

**Published:** 2026-01-11

**Authors:** Yuta Aizawa, Jun Tachikawa, Rie Habuka, Nur Irma Safitri, Ryohei Izumita, Satoshi Hasegawa, Kazuhiro Horiba, Masaaki Kitajima, Akihiko Saitoh

**Affiliations:** Niigata University Graduate School of Medical and Dental Sciences, Niigata, Niigata, Japan; Department of Pediatrics , Niigata University Graduate School of Medical and Dental Sciences, Niigata, Japan, Niigata, Niigata, Japan; Niigata University, Niigata, Niigata, Japan; Niigata Univeisty, Niigata, Niigata, Japan; Niigata University, Niigata, Niigata, Japan; Niigata Prefectural Office, Niigata, Niigata, Japan; Japan Institute for Health Security, Tokyo, Tokyo, Japan; The University of Tokyo, Tokyo, Tokyo, Japan; Niigata University, Niigata, Niigata, Japan

## Abstract

**Background:**

Parechovirus-A (PeV-A) causes a wide clinical manifestation ranging from asymptomatic infection to severe diseases. Among 19 types, PeV-A type 3 (PeV-A3) causes sepsis and encephalitis in young infants. In contrast, PeV-A5 mainly causes gastrointestinal infections and is rarely implicated in severe diseases in such population. Severe diseases by PeV-A5 were reported in Italy, Australia, and Canada in 2018 and 2019. In Japan, 7 infants and children with fever and/or rash were reported in 2018. Following the COVID-19 pandemic, wastewater surveillance has emerged as a valuable tool for detecting community virus circulation independent of clinical case ascertainment. PeV-A5 has been infrequently detected in wastewater globally, different from frequent detection of PeV-A1 and A3, leaving its community circulation poorly characterized.

**Figure:**
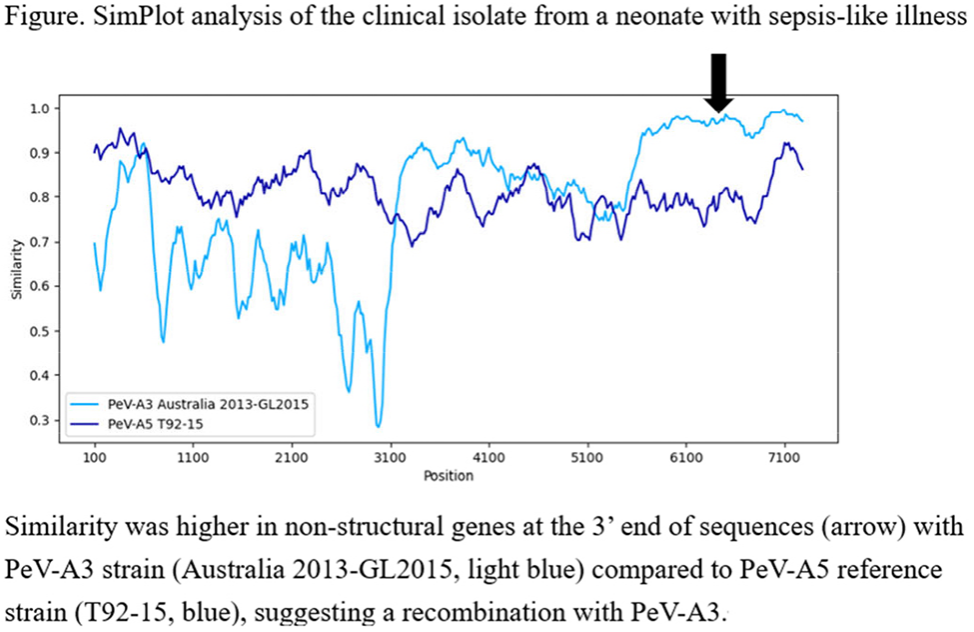

**Methods:**

This is a prospective observational study to identify viruses by collecting serum and cerebrospinal fluid (CSF) samples from febrile infants < 4 months in Niigata, Japan. Simultaneously, we have conducted wastewater surveillance collecting weekly samples from two wastewater treatment plants (WWTP) in Niigata City, Japan. Samples were tested for PeV-A using real-time PCR; positive samples underwent genotyping via Sanger sequencing. The whole genome sequencing (WGS) was performed on the PeV-A isolates from the patient.

**Results:**

Of 33 patients tested in 2024, a single (3.0%) neonate was positive for PeV-A from serum and CSF samples in September. The isolate was genotyped as PeV-A5. The patient resided in a city located to the northeast of Niigata City. Subsequently, PeV-A5 was detected in wastewater samples starting October 2024 from a WWTP serving an area adjacent to the patient's city of residence, and later (November-December 2024) from the other WWTP in Niigata City. WGS analysis of the clinical isolate revealed it to be a recombinant virus, possessing PeV-A5 structural genes and PeV-A3 non-structural genes.

**Conclusion:**

Our findings suggested that a recombinant PeV-A5, potentially acquiring virulence through recombination with PeV-A3, caused sepsis-like illness in a neonate and circulated within the community. Wastewater surveillance provided evidence for the community spread of this virus.

**Disclosures:**

Masaaki Kitajima, Dr.Eng., AdvanSentinel Inc.: Grant/Research Support|Shimadzu Corporation: Grant/Research Support|Shionogi & Co., Ltd.: Grant/Research Support|Shionogi & Co., Ltd.: Receive patent royalties .

